# Supplementary material for: MetaRibo-Seq measures translation in microbiomes
Source: Nat Commun. 2020 Jun 29;11:3268. doi: 10.1038/s41467-020-17081-z (PMC7324362; doi:10.1038/s41467-020-17081-z)
Supplement: Supplementary file 10 — Supplementary Data 7 [file 41467_2020_17081_MOESM10_ESM.zip › File2/Confidence_High_Taxonomy/425534_out.krona.html]

Javascript must be enabled to view this page.

members
magnitude
magnitudeUnassigned
count
unassigned
taxon
rank

425534\_out

314

2
superkingdom
314

976
phylum
314

class
314
200643

171549
order
314

314
family
815

19
816

SRS014235\_contig\_number\_25877SRS015431\_contig\_number\_50708SRS017103\_contig\_number\_27301SRS019910\_contig\_number\_23185SRS022137\_contig\_number\_14481SRS023526\_contig\_number\_25162SRS024075\_contig\_number\_525SRS024625\_contig\_number\_3252SRS044535\_contig\_number\_contig-100\_976.97585SRS047014\_contig\_number\_10709SRS049959\_contig\_number\_49267SRS050299\_contig\_number\_13629SRS051882\_contig\_number\_11981SRS075878\_contig\_number\_22553SRS077335\_contig\_number\_3300SRS077552\_contig\_number\_15118SRS097889\_contig\_number\_14856SRS144362\_contig\_number\_29418SRS893378\_contig\_number\_18652
314
genus

1
species

SRS146764\_contig\_number\_4196
204516

28116

SRS015782\_contig\_number\_5285SRS046369\_contig\_number\_943SRS053398\_contig\_number\_14193SRS104400\_contig\_number\_42048
4
species

329854

SRS018623\_contig\_number\_364
1
species

species
1

SRS045645\_contig\_number\_1037
2292941


SRS014979\_contig\_number\_28073
1
species
246787

species
1

SRS098827\_contig\_number\_194
1262750

46506

SRS016954\_contig\_number\_13414SRS019381\_contig\_number\_453SRS074670\_contig\_number\_1145SRS101376\_contig\_number\_11217SRS104912\_contig\_number\_3698SRS147039\_contig\_number\_494SRS148817\_contig\_number\_228
7
species

5
species

SRS017821\_contig\_number\_25182SRS065504\_contig\_number\_12841SRS077127\_contig\_number\_13621SRS077392\_contig\_number\_37457SRS144135\_contig\_number\_29826
357276


SRS147977\_contig\_number\_1299
species
1
29523

1638779
1
species

SRS050520\_contig\_number\_415

species
3

SRS014855\_contig\_number\_3434SRS017247\_contig\_number\_1136SRS098644\_contig\_number\_30046
817

1
species

SRS013687\_contig\_number\_33710
671267

2292944

SRS015486\_contig\_number\_196SRS049995\_contig\_number\_3664SRS143991\_contig\_number\_15053
species
3

1
species

SRS013158\_contig\_number\_contig-100\_413.40430
371601

376805

SRS054059\_contig\_number\_5187SRS063813\_contig\_number\_2348
species
2

1739298

SRS013521\_contig\_number\_11091SRS078176\_contig\_number\_1638
species
2

821
250
species

SRS011084\_contig\_number\_20773SRS011134\_contig\_number\_32327SRS011239\_contig\_number\_13253SRS011302\_contig\_number\_446SRS011405\_contig\_number\_11285SRS012902\_contig\_number\_199SRS012969\_contig\_number\_31695SRS013382\_contig\_number\_413SRS013638\_contig\_number\_13586SRS013951\_contig\_number\_40523SRS013965\_contig\_number\_29805SRS014287\_contig\_number\_10570SRS014313\_contig\_number\_597SRS014412\_contig\_number\_18942SRS014459\_contig\_number\_24087SRS014534\_contig\_number\_3SRS014683\_contig\_number\_14172SRS014736\_contig\_number\_197SRS015190\_contig\_number\_203SRS015217\_contig\_number\_25492SRS015264\_contig\_number\_17418SRS015578\_contig\_number\_377SRS015663\_contig\_number\_23996SRS015694\_contig\_number\_13127SRS015960\_contig\_number\_12854SRS016056\_contig\_number\_87SRS016095\_contig\_number\_21036SRS016132\_contig\_number\_1119SRS016203\_contig\_number\_2864SRS016335\_contig\_number\_15761SRS016629\_contig\_number\_945SRS016753\_contig\_number\_5939SRS016840\_contig\_number\_2021SRS016897\_contig\_number\_contig-100\_301.302SRS017375\_contig\_number\_461SRS017433\_contig\_number\_2746SRS017521\_contig\_number\_18721SRS017701\_contig\_number\_10249SRS017859\_contig\_number\_2806SRS018351\_contig\_number\_1881SRS018427\_contig\_number\_13577SRS018817\_contig\_number\_12658SRS018836\_contig\_number\_2286SRS018936\_contig\_number\_11272SRS019030\_contig\_number\_10849SRS019068\_contig\_number\_24910SRS019178\_contig\_number\_806SRS019267\_contig\_number\_382SRS019397\_contig\_number\_748SRS019445\_contig\_number\_24790SRS019496\_contig\_number\_14809SRS019601\_contig\_number\_22014SRS019693\_contig\_number\_3766SRS019808\_contig\_number\_19701SRS019968\_contig\_number\_14135SRS020328\_contig\_number\_9643SRS020508\_contig\_number\_257SRS020622\_contig\_number\_1425SRS021153\_contig\_number\_275SRS021219\_contig\_number\_986SRS021484\_contig\_number\_24421SRS021948\_contig\_number\_24343SRS022524\_contig\_number\_15938SRS022713\_contig\_number\_381SRS023829\_contig\_number\_138SRS023914\_contig\_number\_3275SRS024009\_contig\_number\_7288SRS024331\_contig\_number\_20357SRS024492\_contig\_number\_11507SRS042284\_contig\_number\_2335SRS042966\_contig\_number\_19249SRS043411\_contig\_number\_251SRS043667\_contig\_number\_6120SRS043701\_contig\_number\_12092SRS045004\_contig\_number\_2204SRS045244\_contig\_number\_694SRS046068\_contig\_number\_1501SRS046502\_contig\_number\_contig-100\_142.82772SRS046810\_contig\_number\_contig-100\_39.40SRS047433\_contig\_number\_7718SRS048164\_contig\_number\_659SRS049402\_contig\_number\_4077SRS049446\_contig\_number\_2810SRS049712\_contig\_number\_17814SRS049773\_contig\_number\_33420SRS049896\_contig\_number\_26372SRS050026\_contig\_number\_8148SRS050752\_contig\_number\_28607SRS050801\_contig\_number\_contig-100\_288.28335SRS050941\_contig\_number\_6570SRS050974\_contig\_number\_1138SRS050998\_contig\_number\_16477SRS051610\_contig\_number\_624SRS052576\_contig\_number\_172SRS052697\_contig\_number\_41SRS053087\_contig\_number\_2623SRS053214\_contig\_number\_3158SRS053356\_contig\_number\_44579SRS053649\_contig\_number\_14117SRS054905\_contig\_number\_20799SRS055017\_contig\_number\_20477SRS056259\_contig\_number\_12890SRS056273\_contig\_number\_20674SRS056537\_contig\_number\_40143SRS057480\_contig\_number\_9200SRS058268\_contig\_number\_9SRS058723\_contig\_number\_260SRS062427\_contig\_number\_754SRS062654\_contig\_number\_492SRS062701\_contig\_number\_2850SRS063190\_contig\_number\_3310SRS063370\_contig\_number\_1650SRS063518\_contig\_number\_24170SRS063928\_contig\_number\_45SRS064973\_contig\_number\_205SRS065176\_contig\_number\_13629SRS065397\_contig\_number\_4816SRS074586\_contig\_number\_3948SRS074964\_contig\_number\_147SRS075078\_contig\_number\_24691SRS075398\_contig\_number\_246SRS075716\_contig\_number\_694SRS075773\_contig\_number\_389SRS075821\_contig\_number\_919SRS075984\_contig\_number\_20808SRS076756\_contig\_number\_9673SRS076876\_contig\_number\_4SRS076976\_contig\_number\_3575SRS077194\_contig\_number\_11554SRS077231\_contig\_number\_20982SRS077294\_contig\_number\_2752SRS077502\_contig\_number\_12016SRS077589\_contig\_number\_836SRS077641\_contig\_number\_23407SRS077730\_contig\_number\_9554SRS077991\_contig\_number\_610SRS078242\_contig\_number\_8799SRS078665\_contig\_number\_6804SRS097920\_contig\_number\_22178SRS097958\_contig\_number\_13889SRS098061\_contig\_number\_12261SRS098571\_contig\_number\_49199SRS098881\_contig\_number\_15626SRS100021\_contig\_number\_2280SRS100907\_contig\_number\_7887SRS101433\_contig\_number\_12225SRS103987\_contig\_number\_1424SRS104036\_contig\_number\_5378SRS104084\_contig\_number\_2442SRS1041031\_contig\_number\_22500SRS1041032\_contig\_number\_16300SRS1041037\_contig\_number\_7332SRS1041039\_contig\_number\_114SRS1041090\_contig\_number\_5148SRS1041091\_contig\_number\_8626SRS1041092\_contig\_number\_1747SRS1041095\_contig\_number\_820SRS1041116\_contig\_number\_7490SRS1041129\_contig\_number\_6762SRS1041130\_contig\_number\_157SRS1041133\_contig\_number\_21695SRS1041134\_contig\_number\_13958SRS1041137\_contig\_number\_15990SRS1041138\_contig\_number\_1979SRS1041139\_contig\_number\_1978SRS1041140\_contig\_number\_contig-100\_48.118986SRS1041141\_contig\_number\_9037SRS1041142\_contig\_number\_1359SRS1041147\_contig\_number\_525SRS1041157\_contig\_number\_1129SRS1041164\_contig\_number\_638SRS104327\_contig\_number\_6185SRS104485\_contig\_number\_120SRS104693\_contig\_number\_419SRS104975\_contig\_number\_484SRS105082\_contig\_number\_9923SRS105153\_contig\_number\_4428SRS1054716\_contig\_number\_10789SRS1054928\_contig\_number\_1499SRS1055022\_contig\_number\_12365SRS1055038\_contig\_number\_1521SRS1055049\_contig\_number\_993SRS1055056\_contig\_number\_11847SRS1055076\_contig\_number\_2638SRS1055083\_contig\_number\_227SRS1055095\_contig\_number\_410SRS140492\_contig\_number\_16239SRS140513\_contig\_number\_13302SRS142503\_contig\_number\_28187SRS142542\_contig\_number\_504SRS142599\_contig\_number\_3191SRS142781\_contig\_number\_115SRS142890\_contig\_number\_19214SRS142923\_contig\_number\_427SRS142980\_contig\_number\_1691SRS143070\_contig\_number\_12273SRS143085\_contig\_number\_1810SRS143148\_contig\_number\_1732SRS143342\_contig\_number\_1891SRS143372\_contig\_number\_7096SRS143417\_contig\_number\_960SRS143722\_contig\_number\_3558SRS143876\_contig\_number\_601SRS143876\_contig\_number\_23577SRS143895\_contig\_number\_40610SRS144183\_contig\_number\_10224SRS144297\_contig\_number\_12549SRS144506\_contig\_number\_9566SRS144537\_contig\_number\_15467SRS144603\_contig\_number\_13720SRS144714\_contig\_number\_30170SRS144753\_contig\_number\_11856SRS145308\_contig\_number\_117SRS145497\_contig\_number\_1146SRS146813\_contig\_number\_15888SRS147022\_contig\_number\_522SRS147271\_contig\_number\_101SRS147377\_contig\_number\_397SRS147425\_contig\_number\_8097SRS147614\_contig\_number\_1202SRS148091\_contig\_number\_467SRS148196\_contig\_number\_20380SRS148253\_contig\_number\_278SRS148424\_contig\_number\_27551SRS148970\_contig\_number\_765SRS149075\_contig\_number\_12091SRS149181\_contig\_number\_31355SRS149244\_contig\_number\_457SRS149325\_contig\_number\_282SRS892882\_contig\_number\_11157SRS893172\_contig\_number\_10209SRS893187\_contig\_number\_2471SRS893252\_contig\_number\_4853SRS893253\_contig\_number\_539SRS893259\_contig\_number\_6503SRS893270\_contig\_number\_25127SRS893279\_contig\_number\_496SRS893285\_contig\_number\_707SRS893288\_contig\_number\_contig-100\_706.37289SRS893292\_contig\_number\_7152SRS893295\_contig\_number\_12257SRS893327\_contig\_number\_4415SRS893341\_contig\_number\_1550SRS893342\_contig\_number\_674SRS893358\_contig\_number\_281SRS893366\_contig\_number\_15068SRS893380\_contig\_number\_656SRS893382\_contig\_number\_2680SRS893383\_contig\_number\_25386SRS971275\_contig\_number\_32220

818
species
3

SRS015854\_contig\_number\_143SRS077086\_contig\_number\_13246SRS893373\_contig\_number\_16787

291644
species
2

SRS045739\_contig\_number\_6774SRS098073\_contig\_number\_1673

2
species

SRS047044\_contig\_number\_4763SRS051031\_contig\_number\_1259
310298

2
species

SRS077454\_contig\_number\_13091SRS147346\_contig\_number\_12175
47678

1
species

SRS148721\_contig\_number\_17494
1739517
